# Substantiating efficacy of probiotics

**DOI:** 10.1186/2045-7022-1-S1-S8

**Published:** 2011-08-12

**Authors:** Annick Mercenier

**Affiliations:** 1 Nestlé Research Center, Nutrition & Health Department, Lausanne, Switzerland

## 

Even if over 600 probiotic human intervention trials have been performed, not all health promoting effects attributed to probiotics have been substantiated or supported by well conducted clinical trials as yet. However, in evaluating the success met in the area or the validity of the results reached so far, several points should be kept in mind. The targeted health benefits are multiple and diverse, and they deal with populations as different as healthy or at risk infants, adults or elderly. Quite often, the studied disease corresponds to a syndrome rather than a single/simple manifestation from which the underlying causes or drivers are not fully understood. As a consequence, recognized disease risk factors and validated biomarkers are lacking and this hampers fulfilling the present requirements of regulatory authorities. In addition, key questions such as determining the right window of intervention have not always been addressed. While this might not be too difficult to assess when aiming at reducing the risk of diarrhea f.ex. it is quite challenging in the case of allergic diseases: when to optimally intervene in life? When to intervene in the disease course? An additional level of complexity comes from the huge diversity of probiotic candidate strains while it is altogether proven that purported health benefits are strain specific. This renders selection of the best probiotic candidate rather difficult especially since the mechanisms of action of probiotic strains in the highly complex ecosystem(s) they act on has only started to be unraveled. Therefore, dedicated screening strategies are requested. The steps and gaps that need to be filled in order to better substantiate probiotic effects and try to meet the challenge of conciliating science/research and regulation are being evaluated in different working groups such as the ILSI Probiotic Task Force.

